# Electrochemical Sensor Platform for Rapid Detection of Foodborne Toxins

**DOI:** 10.3390/bios15060361

**Published:** 2025-06-04

**Authors:** Kundan Kumar Mishra, Krupa M. Thakkar, Vikram Narayanan Dhamu, Sriram Muthukumar, Shalini Prasad

**Affiliations:** 1 Department of Bioengineering, University of Texas at Dallas, Richardson, TX 75080, USA; kundan.mishra@utdallas.edu (K.K.M.); krupa.thakkar@utdallas.edu (K.M.T.); 2 EnLiSense LLC, 1813 Audubon Pondway, Allen, TX 75013, USA; vikram@enlisense.com (V.N.D.); sriramm@enlisense.com (S.M.)

**Keywords:** electrochemical impedance spectroscopy, toxins, zearalenone, immunosensor

## Abstract

Zearalenone (ZEA), a potent mycotoxin commonly found in contaminated grains, presents a serious threat to food safety and public health. Conventional detection methods, including culture-based assays and laboratory-bound analytical tools, are often time-consuming, require specialized infrastructure, and lack portability, limiting their utility for rapid, on-site screening. In response, this study introduces a compact, real-time electrochemical sensing platform for the swift and selective detection of ZEA in corn flour matrices. Utilizing a non-faradaic, label-free approach based on Electrochemical Impedance Spectroscopy (EIS), the sensor leverages ZEA-specific antibodies to achieve rapid detection within 5 min. The platform demonstrates a low detection limit of 0.05 ng/mL, with a broad dynamic range from 0.1 ng/mL to 25.6 ng/mL. Reproducibility tests confirm consistent performance, with both inter- and intra-assay variation remaining under a 20% coefficient of variation (%CV). Comparative evaluation with standard benchtop systems underscores its accuracy and field applicability. This portable and user-friendly device provides a powerful tool for real-time mycotoxin monitoring, offering significant potential for improving food safety practices and enabling point-of-need testing in resource-limited settings.

## 1. Introduction

Mycotoxins are secondary metabolites produced by mold-forming fungi. Mycotoxins prosper in moisture-rich, humid environments in optimal climate conditions of 25 °C to 35 °C. Additionally, mycotoxin contamination can occur in crops during the initial stages of farming, collecting, storage, and processing [1]. Although over 300 types of mycotoxins have been identified, only a handful have been accurately detected, such as Aflatoxins (AFs), Zearalenone (ZEA), Ochratoxins (OTAs), Fumonisins (FBs), Deoxynivalenol (DON), Patulin, and Trichothecenes. The presence of ZEA in food and feed has become a major concern because it is rapidly absorbed into the bloodstream after ingestion, leading to numerous life-threatening illnesses in both humans and animals [2]. ZEA also accumulates in the food chain, increasing its toxicity. It is a non-steroidal estrogenic mycotoxin produced by the diverse fungi of the Fusarium species, primarily Fusarium culmorum and Fusarium graminearum. Moreover, ZEA expresses distinctive luminescent characteristics under ultraviolet (UV) light. It reveals a radiant green glow at short wavelengths, specifically at 260 nm, and turquoise at longer excitation wavelengths. ZEA is mostly identified in maize, wheat, barely, rice, sorghum, rye, sesame seeds, and hay. Moreover, when animals, such as cows and chickens, consume feed contaminated with ZEA, it can be detected in their byproducts [3,4,5].

ZEA is a xenoestrogen—an exogenous chemical that has potent estrogenic effects due to its structural resemblance to naturally occurring estrogen. ZEA is acknowledged as an endocrine disruptor because it can bind to alpha and beta estrogen receptors. This can lead to the disturbance of many regulatory mechanisms of the endocrine system such as hormone production, secretion, transfer, metabolism, and absorption. With this, disruptions in regard to growth, reproduction, and the maintenance of homeostasis can arise. In females, ZEA poisoning can lead to early puberty, infertility, and pregnancy complications such as decreased embryo viability, reduced fetal weight, and minimal production of milk after childbirth. The dysregulation of estrogen can create abnormalities in the uterine tissue and an imbalance in LH (Luteinizing Hormone) and progesterone, which are hormones essential for menstruation, ovulation, and pregnancy. In males, increased levels of ZEA can impair spermatogenesis, cause oligospermia, and reduce the quality and motility of sperm. ZEA toxicity has severe effects on the liver, such as affecting processes concerning blood coagulation and altering blood markers. In addition, notable amounts of ZEA can lead to the development of liver cancer. ZEA is characterized as a Group III carcinogen by the International Agency for Research on Cancer (IARC). Therefore, the European Union (EU) and Federal Food and Drug Administration (FDA) in the United States have established strict regulations on safe amounts of ZEA in food products sold to the population [6,7,8]. The EU has set a maximum of 20 µg/kg for Zearalenone present in processed cereal-based foods for infants and young children, while the EU requires no more than 100 µg/kg for unprocessed maize intended for human consumption [9,10,11,12], as described in Supplementary Table S1.

Multiple detection methods have been developed to address concerns regarding ZEA contamination in food and feed. Current methods for ZEA detection include enzyme-linked immunosorbent assay (ELISA) [5,13], lateral flow immunoassay (LFIA), polymerase chain reaction (PCR), ultraviolet (UV), and various chromatographic methods, which include gas chromatography (GS), thin-layer chromatography (TLC), and high-performance liquid chromatography (HPLC) with fluorescence (FLD) [5,14]. Although effective, these techniques are exhaustive, complicated, and expensive. They require specialized machinery and trained laboratory technicians to interpret the results. The chromatographic methods are time-consuming because they require complicated steps during preparation, such as using organic solvents for extraction or column set-up. Compared to the methods listed, ELISA is known to provide quick results; however, it does fail to reach the required sensitivity requirements. In addition, both ELISA and PCR require the isolation of the pathogen from the sample, which can be complicated for on-field deployment. While other conventional techniques such as bioluminescence assays, fluorosensors, ELISA with fluorescence, chronoamperometry, and cyclic voltammetry have demonstrated good sensitivity for detecting ZEA, they do possess significant limitations. These include complex and time-consuming sample preprocessing, reliance on labeling agents, longer assay durations, and the requirement for skilled personnel and sophisticated laboratory equipment. Furthermore, many of these methods are designed for single-analyte detection, which adds to the overall cost and time when screening for multiple toxins. Such limitations make these methods laborious and impractical for field use, while raising the issue of cross-contamination that may compromise data accuracy. As summarized in Supplementary Table S2, most existing label-free strategies are not directly sensitive to Zearalenone. These challenges highlight the pressing need for simplified, rapid, and multiplexed electrochemical sensing platforms that are suitable for on-site testing and accessible to non-experts, offering a more practical solution for effective mycotoxin detection.

Given the numerous consequences of ZEA intoxication, it is imperative to develop a detection method that is fast, specific, and practical for on-site use. This proposed method should not require complex and costly laboratory equipment. The sensor must feature a resilient framework that can assure its dependability and robustness for field deployment [15,16,17,18,19,20,21]. Our sensor utilizes Electrochemical Impedance Spectroscopy (EIS), a technique that effectively addresses many limitations associated with traditional toxin detection methods. Conventional approaches such as ELISA, HPLC, and mass spectrometry are well-established and highly accurate but often require complex sample preparation, costly instrumentation, long processing times, and trained personnel, making them less practical for rapid or field-based applications. In contrast, our EIS-based sensor offers a label-free, real-time detection approach that eliminates the need for additional reagents or labeling steps. This significantly reduces both cost and analysis time, while enhancing reliability. The sensor employs surface-modified electrodes functionalized with specific antibodies and a crosslinker to improve target selectivity, signal stability, and reproducibility. We specifically utilize non-faradaic EIS, which monitors impedance changes at the electrode–solution interface without inducing redox reactions. When a target toxin binds to the antibody on the electrode surface, it alters the dielectric properties, leading to detectable changes in the impedance signal. This approach provides several advantages: it is label-free, enables real-time monitoring, offers high sensitivity even at low analyte concentrations, and requires minimal sample preparation. Our findings demonstrate that the developed sensor performs on par with, or better than, traditional laboratory-based methods in terms of sensitivity and specificity. Moreover, the system is compact, low-cost, and user-friendly, eliminating the need for laboratory infrastructure or trained technicians. We validated the sensor using a portable impedance analyzer, which produced results comparable to those obtained from a benchtop potentiostat, further supporting its suitability for use in resource-limited or field settings. Our method integrates the inherent advantages of non-faradaic EIS with a robust and thoughtfully engineered sensor design. This makes it a compelling solution for food safety surveillance and environmental toxin monitoring, particularly in remote or underserved areas.

## 2. Materials and Methods

### 2.1. Materials and Reagents

The ZEA antibody used for sensor functionalization was procured from Invitrogen (Minneapolis, MN, USA). The crosslinker DTSSP (3,3′dithiobis (sulfosuccinimidyl propionate)) and phosphate-buffered saline (PBS) at a pH of 7.4 were obtained from Thermo Fisher Scientific Inc. (Waltham, MA, USA). Samples were divided into small portions that were preserved at −20 °C for future use. Before experimentation, the samples were thawed to room temperature and thoroughly centrifuged to guarantee optimal conditions for testing. Only high-purity, research-grade chemicals were used to bypass additional purification procedures.

### 2.2. ATR-IR Spectroscopy Setup

The Nicolet iS-50 FTIR Spectrometer (Thermo Fisher Scientific Inc., Madison, WI, USA) in Attenuated Total Reflectance mode was used to obtain infrared spectra (IR) data. The spectral window employed Potassium Bromide (KBr), and the sensor utilized Deuterated Triglycine Sulfate (DTGS). A germanium crystal was utilized for spectral analysis to achieve an exceptional resolution of 4 cm^−1^ along a wavelength range of 4000 cm^−1^ to 400 cm^−1^, with a total of 256 scans per cycle. Gold (Au) was applied over the glass substrate surface, followed by the application of zinc oxide (ZnO) for sensor fabrication. Then, DTSSP was used for the modification of the electrode surface, consistent with the immunoassay protocol. After, ZEA antibodies, suspended in PBS, were incubated on the electrode surface.

### 2.3. Modification of Electrode Sensing Platform

To remove potential contaminants that could hinder the detection process, the surface of the electrode was diligently washed with PBS. A solution of 6 mM of DTSSP was blended with 10 μg/mL ZEA antibodies. This solution was incubated in a controlled, supervised environment at 4 °C for 30 min to ensure that light will not interfere with the binding process. Thereafter, the solution was precisely coated across the electrode platform and left to incubate under aluminum foil at room temperature for 30 min. This process affirmed that the solution adequately adhered to the electrode surface. After incubation, the solution was aspirated, and the electrode platform was washed with PBS to remove leftover reagents. Subsequently, superblock was applied to the surface of the electrodes and left to incubate for 10 min away from any light source. Then, the superblock was removed, and the electrode surface was rinsed with PBS. To preserve the durability and integrity of the sensor, the chips were placed in a Lyophilization Machine to freeze to −18 °C, following vacuum pressurization at 5 pascals for 25 min. Upon the completion of the modification procedure, 5 μL of the ZEA antigen was conscientiously spread across each electrode. The sensor was kept in incubation for 5 min to facilitate optimal interaction between the antigen and the antibody. After, EIS was used to analyze the interaction occurring between the sample and electrode. The impedance measurements were conducted with a 10 mV AC bias over a frequency band of 1000 Hz to 80 Hz. This sensor modification procedure delivered essential insights pertaining to the electrochemical properties of ZEA.

### 2.4. Statistical Analysis

The measurements recorded included mean values in conjunction with the standard error of the mean (SEM) to quantify the validity of the recorded data. Furthermore, the data were obtained from three duplicate sensors (*n* = 3). To adhere to the Clinical and Laboratory Standards Institute (CLSI) requirements, both the inter-assay and intra-assay discrepancies were less than 10%. Equivalent circuit study and data modeling was completed through ZView Software (Version 4). Quantitative analysis of the recorded data was carried out by GraphPad Prism (Version 8.01), created by GraphPad Software (La Jolla, CA, USA). The creative designs of the reaction pathway graphics were made using BioRender (BioRender.com, January 2025).

## 3. Results and Discussion

### 3.1. Characterization of the Sensor Platform

**Figure 1:**
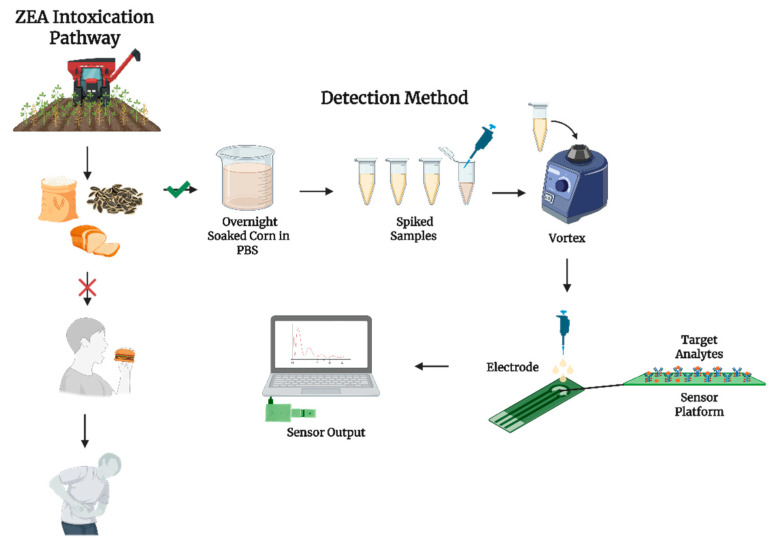
Schematic illustration of corn matrix preparation and electrochemical detection of ZEA using the modified sensor platform.

**Figure 2:**
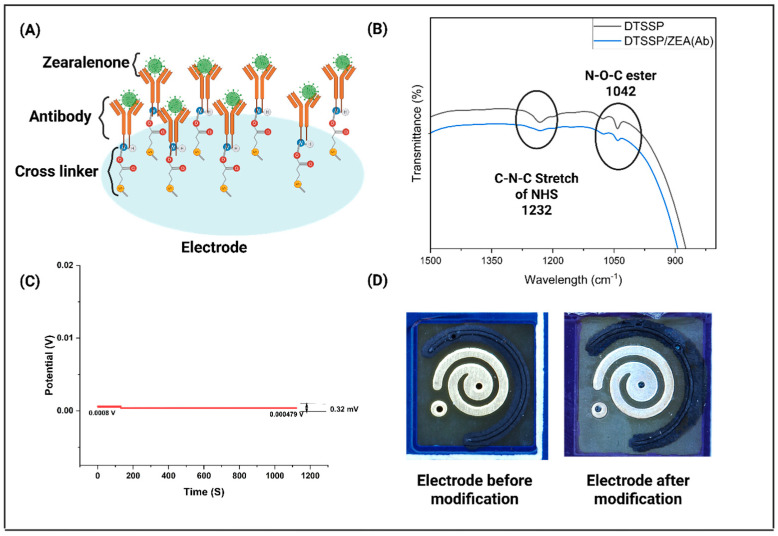
(**A**) A schematic representation of the antibody–antigen interaction mechanism used for the selective detection of Zearalenone. (**B**) FTIR spectra showing (i) DTSSP modification on the ZnO surface and (ii) successful conjugation of Zearalenone antibodies to the DTSSP linker. (**C**) Open circuit potential (OCP) analysis demonstrating the stability of the sensor platform over a period of 1200 s. (**D**) Hirox optical images highlighting the sensor surface prior to and following chemical modification.

In this study, we employed an affinity-based functionalization strategy for the detection of ZEA. The first step in our approach involved the chemical binding of the thiol-based crosslinker, DTSSP [22], to the electrode surface. Subsequently, the ZEA antibody was immobilized onto the surface of the DTSSP crosslinker, as shown in Figure 1. This functionalization strategy utilized affinity-based detection as the primary mechanism for recognizing ZEA. To confirm the successful binding of the crosslinker and antibody, we employed Fourier-Transform Infrared Spectroscopy (FTIR). This verified the crosslinker’s attachment to the electrode and immobilization of the ZEA antibody. To prepare the sensor for FTIR analysis, a thin layer of ZnO was first deposited onto the glass surface via vapor deposition. Then, the ZnO surface was modified with the ZEA antibody to create the sensor platform. The addition of ZnO on the sensor surface significantly enhanced the interactions between the antibody and the target analyte, thereby improving the sensitivity of the sensor. This modification also strengthened the interactions occurring on the surface of the sensor, ultimately improving the detection performance. The FTIR results corroborated the successful immobilization of the antibody, with a characteristic peak observed at 1742 cm^−1^ after surface modification with DTSSP [23,24,25,26,27]. Additional peaks were identified at 1232 cm^−1^, corresponding to the C-N-C stretch of N-hydroxysuccinimide (NHS), and at 1042 cm^−1^, associated with the N-O-C esters group, as shown in Figure 2B. The disappearance of these peaks, namely at 1742 cm^−1^, 1232 cm^−1^, and 1042 cm^−1^, indicated the successful formation of amide bonds between the DTSSP crosslinker and the antibody on the ZnO surface, confirming the effective immobilization process [28]. To further evaluate the performance of the fabricated sensor, we examined its stability using open circuit potential (OCP) measurements. Figure 2C presents the OCP data of the sensor platform over a 1200 s duration for ZEA detection, demonstrating minimal fluctuations in the sensor’s potential, with a change of only 0.32 mV. This stable response is crucial for ensuring the accuracy and reliability of toxin detection over extended periods, thus enhancing the sensor’s potential for long-term, practical application. The minimal potential variations highlight the sensor’s robustness, confirming its ability to provide consistent and reliable data for toxin monitoring in diverse sample environments. Moreover, the optical images of the sensor platform, depicted in Figure 2D, visually confirm the sensor’s design and surface characteristics, further supporting the successful implementation of the sensor for practical detection purposes.

### 3.2. Electrochemical Signal Response on the Modified Sensor Platform and Spike and Recovery Study

**Figure 3:**
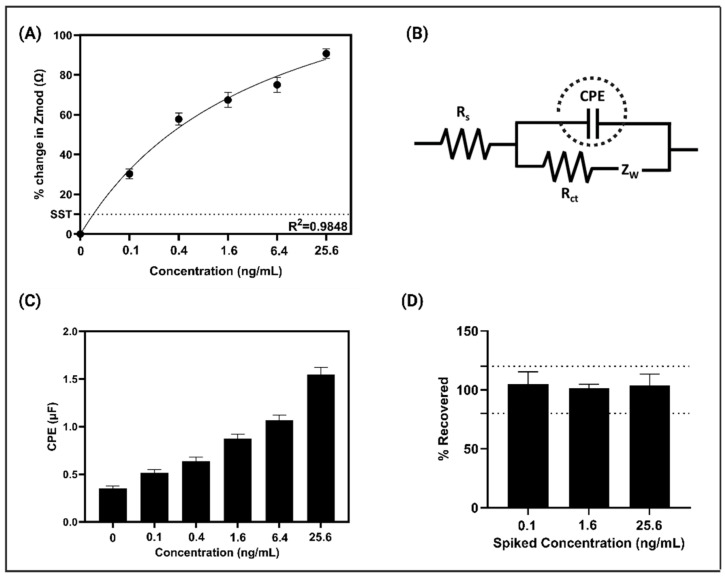
(**A**) A calibration dose–response (CDR) curve illustrating the detection of Zearalenone across concentrations from 0.1 to 25.6 ng/mL in corn samples soaked overnight. (**B**) The corresponding equivalent circuit models used for sensor analysis. (**C**) Changes in electrical double-layer capacitance observed with varying Zearalenone levels in corn samples. (**D**) Recovery plot demonstrating the sensor’s accuracy in detecting different concentrations of Zearalenone.

EIS was utilized to examine the electrochemical interactions occurring on the sensor platform for the detection of ZEA [29]. EIS data were collected by applying a 10 mV AC bias [22,29,30], capturing the voltage changes in the sensor’s double-layer as ZEA antigens bound to the immobilized antibodies on the surface. This method provided a highly sensitive and precise detection mechanism that broadens the sensor’s potential usage for environmental monitoring and medical diagnostics. Impedance data from multiple runs were used to generate Calibrated Dose Response (CDR) curves, which correlated the impedance values with specific antigen concentrations. These curves were developed using overnight soaked corn samples spiked with ZEA (0.1–25.6 ng/mL) that offered a comprehensive evaluation of the sensor’s performance. Figure 3A presents a detailed analysis of dose-dependent impedance plots, demonstrating a strong linear relationship between the percentage change in impedance and ZEA concentrations. The data show a progressive increase in impedance as ZEA concentrations rise, driven by the modulation of the sensor’s double-layer charge. This trend highlights the sensor’s ability to accurately quantify antigen levels. The CDR plot derived from these measurements exhibited an impressive correlation coefficient (R^2^ = 0.9848), confirming the platform’s high sensitivity and precision. The frequency spectrum analysis revealed that 200 Hz was the optimal frequency for generating these plots, as it provided the highest signal-to-noise ratio. To further interpret the electrochemical behavior of the system, the Randles equivalent circuit model was applied (Figure 3B). This model included key electrical components such as solution resistance (Rs) in series with a parallel network consisting of ZnO resistance (RZnO) and ZnO capacitance (CZnO). A secondary parallel RC circuit dominated at lower frequencies, offering deeper insight into the sensor’s electrochemical processes. The charge-transfer resistance (Rct) and constant phase element (CPE) within this circuit indicated non-ideal behavior due to surface roughness and variations in double-layer capacitance. As shown in Figure 3C, a significant increase in CPE values with rising ZEA concentrations highlights the sensor’s high sensitivity to antigen variations. This trend emphasizes the dynamic nature of antigen–antibody interactions, reinforcing the platform’s reliability for detecting ZEA. The thorough analysis validates the sensor’s capability for precise quantification, making it a robust tool for food safety and environmental monitoring.

A spike and recovery study is a critical analytical method used to assess the accuracy and reliability of measurement techniques. In this study, controlled concentrations of ZEA were added to the samples to evaluate the sensor’s performance through percentage recovery calculations using the CDR. This approach provided an evaluation of the detection method’s preciseness by comparing the expected concentrations with those measured by the sensor. This comparison ensured that any deviations or inaccuracies in the measurement process were systematically identified. For ZEA, concentrations ranging from 0.1 ng/mL (low) and 1.6 ng/mL (medium) to 25.6 ng/mL (high) were randomly spiked into overnight soaked corn samples, with the study including *n* = 8 replicates. Furthermore, the sensor’s limit of detection (LoD) was determined using the signal-to-noise (S/N) approach. The specific signal threshold (SST) was defined as the mean baseline signal plus three times the standard deviation. Based on this method, the sensor demonstrated an LoD of 0.05 ng/mL, highlighting its high sensitivity for detecting trace levels of ZEA [23]. Figure 3D shows the percentage recovery plot for the overnight soaked corn matrix, with recoveries consistently remaining below 20% within the given concentration range. This highlights the sensor’s capability to accurately measure the added ZEA concentrations and further supports the sensor’s reliability in quantifying low to high concentrations of the toxin [23].

### 3.3. Cross-Reactivity, Reproducibility, and Repeatability

**Figure 4:**
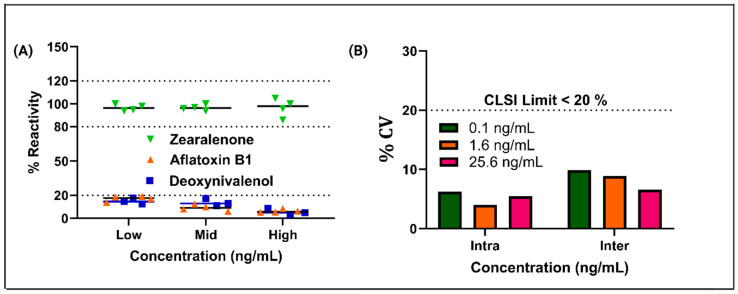
(**A**) A cross-reactivity analysis of the Zearalenone-specific sensor in the presence of Aflatoxin B1 and Deoxynivalenol. (**B**) An evaluation of inter-study and intra-study variability to assess the reproducibility and reliability of the sensor platform.

A sensor intended for field applications must exhibit high selectivity to ensure reliable performance during real-world conditions. In a real-world situation, the presence of various nutrients and matrix complexities can interfere with the binding of essential compounds on the electrode surface. Moreover, structurally similar toxins present in the sample at comparable or higher concentrations can affect the interpretation of the sensor data. To assess the sensor’s specificity, we performed cross-reactivity testing by spiking samples of overnight soaked corn with various cross-reactive toxins at different concentrations of ZEA. Figure 4A presents the response graph for the cross-reactivity study, demonstrating that the ZEA-Ab sensor platform exhibited less than a 20% change in impedance values when exposed to high concentrations of interfering compounds, such as AFB1 and DON. The impedance response from these interfering compounds remained minimal. The ZEA signal showed a distinct and significantly higher response, particularly at 200 Hz, which was the optimal frequency for detection. This minimal cross-reactivity highlighted the sensor’s exceptional specificity, crucial for accurate detection in complex food matrices. The sensor’s ability to selectively detect ZEA while minimizing interference from structurally similar substances further ensured its reliability for food safety monitoring and field-based testing [31]. Figure 4B shows the relationship between the actual concentration and the predicted concentration from the device across low, mid, and high concentration ranges in overnight soaked corn samples. When ZEA levels were low, the device indicated that the food was safe for consumption. However, when concentrations fell within the mid or high range, the device signaled that ZEA levels exceeded the allowable safety limits, highlighting a potential health risk. The sensor’s accuracy and precision for detecting low concentrations of ZEA in overnight-soaked corn samples was validated through comprehensive intra-assay and inter-assay evaluations. Intra-assay repeatability assessed consistency within a single assay. Inter-assay reproducibility ensured reliability across multiple, independent assays. As illustrated in Figure 4B, the coefficient of variation (%CV) that was tested for concentrations of 0.1 ng/mL, 1.6 ng/mL, and 25.6 ng/mL remained consistently below 20%, aligning with the rigorous acceptance criteria set by the Clinical and Laboratory Standards Institute (CLSI) [32]. Additionally, the sensor’s short-term stability was evaluated over a four-week period at 4 °C, with performance monitored weekly. The results, presented in Supplementary Figure S1, demonstrate the sensor’s stable impedance response over time, confirming its robustness and reliability for extended use. This ensures that the sensor is highly reliable and accurate for detecting ZEA in food matrices, reinforcing its potential for real-world application, particularly in food safety and environmental monitoring contexts where the detection of low concentrations of toxins is critical for public health. The consistency of results across different assays further emphasizes the robustness and precision of the sensor platform.

### 3.4. Correlation Study Between the Benchtop and Portable Device

**Figure 5:**
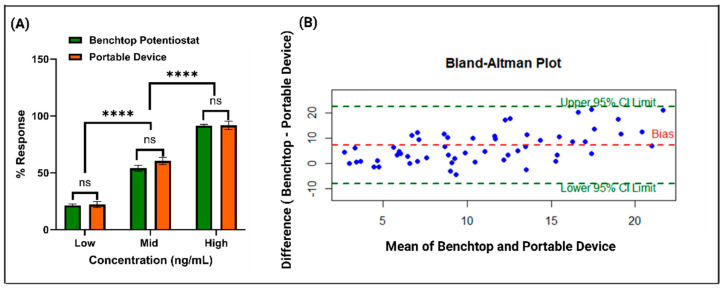
(**A**) A comparative analysis of dose—response measurements obtained from portable and benchtop devices, with statistical evaluation using paired *t*-tests. ‘ns’ denotes no significant difference between matched doses, while ‘****’ indicates statistically significant differences across low-to-mid and mid-to-high concentration ranges for both platforms. (**B**) A Bland–Altman plot assessing agreement between the two measurement systems, showing a mean bias of 8.3.

To further evaluate the performance and practical utility of the developed sensor system, impedance measurements obtained using a benchtop potentiostat and a portable device were compared, as shown in Figure 5A. A paired *t*-test revealed no statistically significant difference between the two instruments for corresponding concentrations of ZEA (*p* = 0.74). This close agreement indicates that the portable device delivers results comparable to those of a laboratory-grade potentiostat, highlighting its potential for field-based toxin detection. Importantly, statistically significant differences were observed between low-to-mid and mid-to-high ZEA concentrations across both platforms (*p* = 0.000123), suggesting that both systems can reliably distinguish between varying concentration ranges. This ability is essential for applications that require the precise quantification of toxin levels to ensure food safety, regulatory compliance, and health risk assessment. Figure 5B presents a Bland–Altman plot, where the *x*-axis denotes the mean impedance value for each ZEA concentration measured from overnight-soaked corn samples, while the *y*-axis shows the difference in impedance readings between the benchtop and portable devices. The analysis revealed a mean bias of 8.3 ohms, indicating only a minor discrepancy between the two systems, particularly at higher toxin concentrations. All data points fell within ±1.96 standard deviations of the mean, establishing a 95% confidence interval and confirming strong agreement between the devices. The symmetrical distribution of data points above and below the bias line further supports the absence of systematic over- or underestimation by either method. Collectively, these findings validate the portable device’s reliability, reproducibility, and accuracy, demonstrating its suitability for scalable deployment in food safety monitoring, especially in low-resource or remote environments with limited access to laboratory infrastructure.

## 4. Conclusions

An electrochemical sensing platform based on Electrochemical Impedance Spectroscopy (EIS) was successfully developed for the detection of Zearalenone in overnight-soaked corn samples. The integration of immunoassay techniques using Zearalenone-specific antibodies enabled high selectivity and accurate detection, minimizing the risk of cross-interference. The platform delivered rapid results within 5 min using only a 5 µL sample volume. It achieved an impressive detection limit of 0.05 ng/mL and demonstrated robust and reproducible performance across a wide concentration range in both laboratory and portable formats. Cross-reactivity evaluations confirmed strong specificity toward Zearalenone, underscoring the platform’s reliability. Due to its portability, user-friendliness, and analytical precision, this electrochemical immunosensor offers a promising solution for the on-site detection of mycotoxins, contributing to improved food safety and public health surveillance.

## Data Availability

The data that support the findings of this study are available on request from the corresponding author. The data are not publicly available due to privacy or ethical restrictions.

## Supplementary Materials

The following supporting information can be downloaded at https://www.mdpi.com/article/10.3390/bios15060361/s1, Figure S1: Stability assessment of the Zearalenone sensor over four weeks of storage at 4 °C; Table S1: Maximum limit of zearalenone by food product as per EU standards; Table S2: Comparison of the developed immunosensor with other studied label-free detection of AB1 and ZEA.
