# Supplementary material for: Electrochemical Sensor Platform for Rapid Detection of Foodborne Toxins
Source: Biosensors (Basel). 2025 Jun 4;15(6):361. doi: 10.3390/bios15060361 (PMC12191336; doi:10.3390/bios15060361)
Supplement: Supplementary file 1 [file biosensors-15-00361-s001.zip › biosensors-3657211-supplementary.pdf]

biosensors

Supplementary

Electrochemical Sensor Platform for Rapid Detection of Food-borne Toxins

Kundan Kumar Mishra [1], Krupa M Thakkar [1], Vikram Narayanan Dhamu [2], Sriram Muthukumar [2] and Shalini Prasad [1,2,]*

[1] Department of Bioengineering, University of Texas at Dallas, Richardson, TX 75080, US
[2] EnLiSense LLC, 1813 Audubon Pondway, Allen, TX 75013, USA
* Correspondence: shalini.prasad@utdallas.edu

Table S1. Maximum limit of zearalenone by food product as per EU standards [1,2.]

Food Product	Maximum Limit (μg/kg)
Unprocessed cereals (not including maize)	100
Cereal Flour (not including maize flour)	75
Bread Pastries and Biscuits	50
Other Cereal Snacks and Breakfast Cereal (not including maize)	50
Processed Cereal Based Foods for Infants and Young Children	20

Table S2. Comparison of the developed immunosensor with other studied label-free detection of AB1 and ZEA.

Device Type/ Detection Method	Mycotoxin Detected	Sample Type	LoD	Assay Time	Reference
Amperometric	Zearalenone	Maize	0.011 μg/L	Not Given	[3]
Square Wave Voltammetry	Zearalenone	Human biofluids	0.005 ng/mL	112 minutes for sample and sensor preparation 20 minutes for reading	[4]
Cyclic Voltammetry	Zearalenone	Amaranthus cruentus seeds	0.57 ng/mL	54 hours for sample and sensor preparation 5 minutes per reading	[5]
Fluorosensor	Zearalenone	Acetonitrile	2.5 × 10−5 M	20.5 hours for sample and sensor preparation Time for reading not given	[6]
Bioluminescence assay	Zearalenone	Milk	1 nM for α-zearalenol	3 hours assay time	[7]
Fluorosensor	Zearalenone	Wheat	0.018 mg/L	17.5 hours for sensor and sample preparation Time for reading not given	[8]

Aptameric with Fluorescence	Zearalenone	Corn Juice	0.085 fg/mL	46+ hours for sensor and sample preparation Time for reading not given	[9]
ELISA with Fluorescence	Zearalenone	Corn	4.1 pg/mL	96.75+ hours for sample preparation Time for reading not given	[10]
Electrochemical Impedance Spectroscopy	Zearalenone	Corn	0.05 ng/mL	Sample Running time 5 Min.	This paper

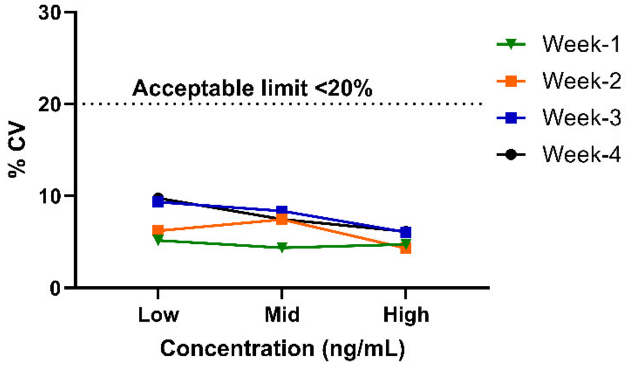

Figure S1. Stability assessment of the Zearalenone sensor over four weeks of storage at 4 °C. The impedance response was measured weekly (Week 1 to Week 4) at low (0.1 ng/mL), mid (1.6 ng/mL), and high (25.6 ng/mL) Zearalenone concentration, showing consistent signal levels with minimal variation, indicating stable sensor performance over time.

References:

1. Boelaert, F.; Van der Stede, Y.; Stoicescu, A.; Amore, G.; Nagy, K.; Rizzi, V.; Felicio, M.T.D.S.; Messens, W.; Pelaez, A.O.; Hempen, M.; et al. The European Union Summary Report on Trends and Sources of Zoonoses, Zoonotic Agents and Food-Borne Outbreaks in 2017. *EFSA Journal* **2018**, *16*, e05500, doi:10.2903/j.efsa.2018.5500.
2. Union, E. COMMISSION REGULATION (EC) No 856/2005 of 6 June 2005 Amending Regulation (EC) No 466/2001 as Regards Fusarium Toxins 2005. https://www.legislation.gov.uk/eur/2005/856.
3. Hervás, M.; López, M.Á.; Escarpa, A. Electrochemical Immunoassay Using Magnetic Beads for the Determination of Zearalenone in Baby Food: An Anticipated Analytical Tool for Food Safety. *Anal Chim Acta* **2009**, *653*, 167–172, https://doi.org/10.1016/j.aca.2009.09.024.
4. Jiang, K.; Nie, D.; Huang, Q.; Fan, K.; Tang, Z.; Wu, Y.; Han, Z. Thin-Layer MoS2 and Thionin Composite-Based Electrochemical Sensing Platform for Rapid and Sensitive Detection of Zearalenone in Human Biofluids. *Biosens Bioelectron* **2019**, *130*, 322–329, doi:10.1016/j.bios.2019.02.003.
5. Regiart, M.; Fernández, O.; Vicario, A.; Villarroel-Rocha, J.; Sapag, K.; Messina, G.A.; Raba, J.; Bertolino, F.A. Mesoporous Immunosensor Applied to Zearalenone Determination in Amaranthus Cruentus Seeds. *Microchem J* **2018**, *141*, 388–394, doi:10.1016/j.microc.2018.05.051.
6. Navarro-Villoslada, F.; Urraca, J.L.; Moreno-Bondi, M.C.; Orellana, G. Zearalenone Sensing with Molecularly Imprinted Polymers and Tailored Fluorescent Probes. *Sens Actuators B Chem* **2007**, *121*, 67–73, doi:10.1016/j.snb.2006.09.042.
7. Välimaa, A.-L.; Kivistö, A.T.; Leskinen, P.I.; Karp, M.T. A Novel Biosensor for the Detection of Zearalenone Family Mycotoxins in Milk. *J Microbiol Methods* **2010**, *80*, 44–48, doi:10.1016/j.mimet.2009.10.017.
8. Du, Q.; Wu, P.; Hu, F.; Li, G.; Shi, J.; He, H. Novel Molecularly Imprinted Polymers on Metal–Organic Frameworks as Sensors for the Highly Selective Detection of Zearalenone in Wheat. *New J Chem* **2019**, *43*, 7044–7050, doi:10.1039/C9NJ00589G.

9. Xiang, S.; Li, J.; Wang, F.; Yang, H.; Jiang, Y.; Zhang, P.; Cai, R.; Tan, W. Novel Ultralow-Potential Electrochemiluminescence Aptasensor for the Highly Sensitive Detection of Zearalenone Using a Resonance Energy Transfer System. *Anal Chem* **2023**, *95*, 15125–15132, doi:10.1021/acs.analchem.3c03437.
10. Zhan, S.; Huang, X.; Chen, R.; Li, J.; Xiong, Y. Novel Fluorescent ELISA for the Sensitive Detection of Zearalenone Based on H2O2-Sensitive Quantum Dots for Signal Transduction. *Talanta* **2016**, *158*, 51–56, doi:10.1016/j.talanta.2016.05.035.
